# Enhanced Piezoelectric Fibered Extracellular Matrix to Promote Cardiomyocyte Maturation and Tissue Formation: A 3D Computational Model

**DOI:** 10.3390/biology10020135

**Published:** 2021-02-09

**Authors:** Pau Urdeitx, Mohamed H. Doweidar

**Affiliations:** 1 Mechanical Engineering Department, School of Engineering and Architecture (EINA), University of Zaragoza, 50018 Zaragoza, Spain; purdeitx@unizar.es; 2 Aragon Institute of Engineering Research (I3A), University of Zaragoza, 50018 Zaragoza, Spain; 3 Biomedical Research Networking Center in Bioengineering, Biomaterials and Nanomedicine (CIBER-BBN), 50018 Zaragoza, Spain

**Keywords:** cardiac muscle tissue, mesenchymal stem cells, cardiomyocyte, 3D in silico modeling, electrotaxis, mechanotaxis

## Abstract

**Simple Summary:**

Cell development for tissue regeneration depends on the mechanical and the electrical stimuli present in the cell microenvironment. This is especially relevant for tissues with complex cellular structures such as cardiac tissue. To recognize the complex interaction of the cell with its microenvironment, it is necessary to understand the role of the mechanical forces generated by the previously mentioned stimuli in the cell behavior. Studying this process, through in vitro models, requires a large number of experiments, with a high economic and time cost. In this sense, computational methods are capable of reproducing cell mechanics within complex microenvironments considering cell–cell and cell–extracellular matrix interactions. Thus, we have developed a 3D computational model to reproduce this process. With this model, different experiments have been purposed to study cardiac cell differentiation and aggregate morphology, under different extracellular matrix configurations. According to the results, group morphologies are determined by the intensity and the directionality of the applied stimuli. Using the developed model, it is possible to develop parametric studies to determine the suitable preliminary conditions for adequate tissue development, reducing the number of in-vitro experiments.

**Abstract:**

Mechanical and electrical stimuli play a key role in tissue formation, guiding cell processes such as cell migration, differentiation, maturation, and apoptosis. Monitoring and controlling these stimuli on in vitro experiments is not straightforward due to the coupling of these different stimuli. In addition, active and reciprocal cell–cell and cell–extracellular matrix interactions are essential to be considered during formation of complex tissue such as myocardial tissue. In this sense, computational models can offer new perspectives and key information on the cell microenvironment. Thus, we present a new computational 3D model, based on the Finite Element Method, where a complex extracellular matrix with piezoelectric properties interacts with cardiac muscle cells during the first steps of tissue formation. This model includes collective behavior and cell processes such as cell migration, maturation, differentiation, proliferation, and apoptosis. The model has employed to study the initial stages of in vitro cardiac aggregate formation, considering cell–cell junctions, under different extracellular matrix configurations. Three different cases have been purposed to evaluate cell behavior in fibered, mechanically stimulated fibered, and mechanically stimulated piezoelectric fibered extra-cellular matrix. In this last case, the cells are guided by the coupling of mechanical and electrical stimuli. Accordingly, the obtained results show the formation of more elongated groups and enhancement in cell proliferation.

## 1. Introduction

The processes of migration, differentiation, proliferation, and apoptosis play a key role in tissue development. These processes are governed by complex mechanical, thermal, electrical, and chemical cues in the extracellular matrix (ECM), which are perceived by the cells through cell–cell and cell–ECM interactions. In the last decades, the interest to understand these processes and the cues which govern them has been increased. Greater knowledge of the main cellular processes, as well as the factors that trigger these processes, can provide new cell therapies such as the induced regeneration of tissues or the manufacture of tissues or organs in laboratories. Among these factors are the mechanical properties of the ECM, which are particularly important in the regulation of these processes. In the late 1990s, it was shown that changes in the tissue mechanical conditions could induce its growth or remodeling. However, in the last two decades, great advances have been made in this field [1,2,3,4,5]. The reciprocal interaction of the cells with their ECM has been seen to be essential for tissue development. Alterations of the ECM conditions trigger specific responses in cells, such as migration, proliferation, differentiation, and apoptosis. In addition, the cell interacts with the ECM altering its shape and composition [5]. This active cell–ECM interaction results in a regulation of tissue architecture, which is closely related to tissue function [6,7]. Engler et al., 2006, observed that the effects of ECM stiffness could define the specification of the stem cell lineage, regulating the process of cell differentiation in different adherent cell types such as neurons, myoblasts, and osteoblasts [8]. Subsequently, other authors have extended this theory, achieving spontaneous differentiation in other cell lineages [9,10,11].

Among these processes, cell migration has presented an increasing interest in the scientific community. Its involvement in cellular processes such as tissue growth and remodeling, together with the possibility of using the patient’s own stem cells, opens the door to new cell therapies. Additionally, a greater knowledge of the stimuli associated with cell migration may offer new perspectives to prevent cancer metastasis [12]. Thus, the description of cell migration processes has been highly expanded recently. The effects of cell–cell interaction [3], cell communication [4], deformations, and cellular adhesions [5], as well as the consideration of new stimuli [13], during migration have been extensively studied. These studies show that the cellular environment, with complex cell–cell and cell–ECM interactions, as well as the coupling of different stimuli, should be considered in the study of different cellular processes. Despite its complexity, cellular therapies have been shown to be effective in tissues that have shown limitations in their regeneration, such as heart tissue.

Currently, cardiovascular diseases are presented as the main cause of death worldwide. The damages caused after a heart attack are not recoverable. With this perspective, different authors have proposed different strategies for the recovery of damaged tissue [14,15,16]. Thus, they have studied the possibility of developing cardiac tissues that could subsequently be implanted [10,17,18,19,20]. An ambitious study was presented by H. C. Ott et al., 2008, in which they presented a novel protocol to create a contractile whole heart from decellularized and repopulated with freshly isolated neonatal cardiac cells [21]. This technique has been extended later by other researchers, obtaining promising results [22]. These interesting proposals could respond to different cardiovascular pathologies in the near future. However, the contractile capacities of the developed cardiac tissues using stem cells seem to be still below those developed in an adult heart. Lack of cell maturity or low tissue innervation seems to be the main cause of this problem [23]. The complex cell–cell and cell–ECM interactions, as well as the stimuli to which the cell is subjected during cardiac cell maturation, make it difficult to understand and adequately control the different cellular processes that, ultimately, control the growth of tissues. To improve cardiac tissue regeneration, it is necessary to establish optimum conditions during cell development, which implies the development of a large number of in vitro experiments. These multiple assays, varying cell culture parameters in a wide range, involve a high time and economic cost. At this point, computational models can offer clear advantages to support in vitro investigations, bringing new conclusions and perspectives to improve tissue development.

Two approaches are commonly adopted in the development of cellular computational models: continuous cellular models, which are based on cellular densities, and discrete cellular models, which is a more detailed approach to each individual cell [24]. Continuum models have been widely used in the study of wound healing [25], the consumption of nutrients [26,27], and the design of scaffolds [28,29], with low computational costs. However, cell–cell and cell–ECM interactions, which are essential for different cell processes, are usually neglected in this perspective. On the other hand, models based on discrete cells have been developed where it is possible to analyze the cellular environment from the perspective of each individual cell. These models can thoroughly consider cell–cell and cell–ECM interactions, as well as the local influence of the different stimuli on cells. Among these models, different approaches can be found in cell migration [30,31,32,33,34], morphology [35,36,37,38], proliferation [39,40], and differentiation [41,42]. However, neither the collective behavior resulting from cardiomyocyte (CM) intercommunication nor the formation of stable cell adhesions has been considered before. These aspects play a key role in collective cell migration, as well as in the cell architecture of the tissues [5]. Thus, we present a discrete CM model to study cell differentiation, migration, proliferation, and apoptosis processes based on electro-mechanical stimulation of the ECM. In this model, we consider the formation of stable cardiac cell adhesions, resulting from the cell–cell interaction, which generates collective behaviors, such as collective migration and the formation of specific cellular architectures.

## 2. Materials and Methods

In this paper, we present a new computational model for cardiac cell behavior in 3D-enhanced piezoelectric fibered ECM. This model includes cell migration, differentiation, proliferation, and apoptosis, as well as the consideration of complex cell–cell and cell–ECM interactions that promote collective cell behavior. This model has been implemented through the Finite Element Method (FEM), using Abaqus user subroutine UELMAT [43]. In the present model, the cell has been defined as a 24-node, quasi-spherical, user-defined element. The cell is surrounded by an enhanced ECM, which is discretized using trilinear hexahedral elements. Along the longitudinal axis, at the center of the ECM, a (piezoelectric) fiber element has been defined, with greater stiffness than the ECM. The calculation is divided into different phases. Initially, to experiment their environment, the cell applies sensing forces on the ECM through the nodes located in the cell membrane. A stress–strain equilibrium is established in the cell–ECM interface from which the cell’s internal deformations are obtained. Then, through these deformations, the internal stresses of the cell are defined, which in turn are used to calculate the motor forces of the cell. In parallel, cell deformations are used to define the level of mechanical stimulation to which the cell is subjected, with which the processes of maturation, differentiation, and apoptosis are controlled. Then, the cells’ global polarization direction is determined as a function of the individual cell polarization direction. When the contact direction between cells is consistent with the global polarization direction, they establish stable cell adhesion. Thus, cardiac cells remain attached and, consequently, collective cell behavior is developed. In this sense, collective cell migration is considered for groups of cardiac cells that are attached with stable cell junctions. Finally, if there is no collective migration, the cells are able to relocate themselves for more favorable locations.

### 2.1. Assumptions

**Figure 1:**
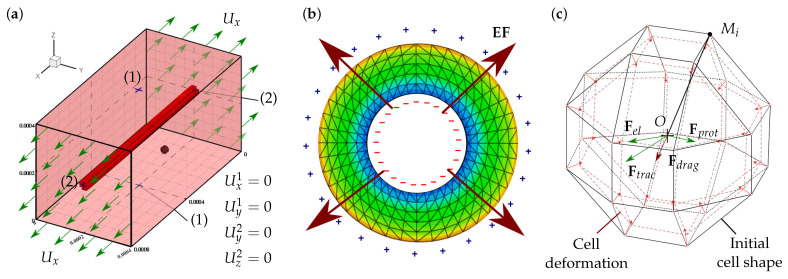
Piezoelectric fibered ECM and cell discretization. (**a**) Description of the fibered ECM configuration with the considered boundary conditions. A piezoelectric fiber, stiffer than the ECM, is located at the center of the ECM. To ensure the calculation stability, the displacements of points (1) and (2) are restricted in such a way that $U_{x}^{1}=U_{y}^{1}=0$ and $U_{y}^{2}=U_{z}^{2}=0$, respectively. Displacements in the *X* direction are imposed on planes $X_{0}$ and $X_{0.0008}$ to produce an ECM strain of 0.25. (**b**) Piezoelectric active response to the imposed deformation on the ECM. Electric field gradient is generated with a positive charge on the external surface. (**c**) Cell discretization with 24 nodes in the cell membrane, on which cell–cell and cell–ECM interaction forces are evaluated. Cell internal deformation is evaluated as the variation of the distance between each membrane node, $M_{i}$, with respect to the cell centroid, *O*.

The model is employed to study CMs behavior in in vitro conditions, where cells are cultured in synthetic 3D ECM under controllable mechanical and electrical conditions (Figure 1). Different considerations have been taken into account in the development of this model. The model considers the cell culture environment as a homogeneous hydrogel, with the necessary conditions for cell survival. For simplicity, the model considers multipotent cell phenotype as mesenchymal stem cell (MSC), and cells of the cardiac lineage as CMs. The cells are considered to have quasi-spherical morphology and maintain their morphology throughout the simulation. In addition, spontaneous MSCs differentiation into CMs induced by a mechanical stimulation is considered. Passive mechanical stimulation due to ECM stiffness and active mechanical stimulation due to external ECM deformation are also considered. The ECM deformation is considered to be applied in the longitudinal direction. In addition, a more rigid longitudinal fiber has been considered within the ECM. When an electric stimulus is considered, piezoelectric properties have been assigned to the internal fiber in the ECM.

### 2.2. Cell Migration

**Figure 2:**
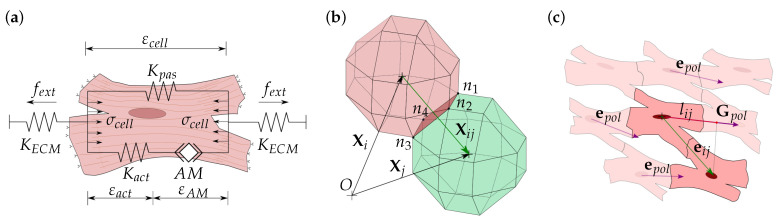
Mechanical modeling of the cell. (**a**) Equivalent mechanical model of the cell. $\sigma_{cell}$ is the cell internal stress generated due to the contraction of the actin–myosin (*AM*) filaments, $\varepsilon_{AM}$, which causes cell internal deformation, $\varepsilon_{cell}$. $K_{act}$ is the active stiffness of the cell generated by the active *AM* apparatus. $K_{pas}$ is the passive stiffness due to cell membrane and cell cytoskeleton resistance. Externally, the cell is attached to the extracellular matrix (ECM), with which the cell interacts by deforming the ECM, generating an opposing force, $f_{ext}$, due to the ECM stiffness, $K_{ECM}$. (**b**) Cell–cell contact vector, $X_{ij}$, which can be calculated through the coordinate vectors $X_{i}$ and $X_{j}$ of the *i*th and *j*th cells, respectively. At any time step, cells in contact could satisfy $\|X_{ij}\|\leq 2r$. Any face in contact, defined by the nodes ($n_{1}:n_{4}$), loses the capacity to interact with the ECM. (**c**) Cardiac cell establishes stable cell–cell interactions, cell junctions (CJs), when the direction of the cell contact vector, $e_{ij}$, is consistent with the cells global polarization direction, $G_{pol}$. $G_{pol}$ is calculated through the individual cell polarization direction, $e_{pol}$. To compare $e_{ij}$ and $G_{pol}$, the projection, $l_{ij}$, is defined for each pair of cells in contact, being considered as a CJ when $l_{ij}\geq l_{min}$, and $\|X_{ij}\|=2r$.

Cell migration depends on the contractile activity of the actin–myosin (*AM*) apparatus [44,45,46]. The cell, through their focal adhesions, interacts with the ECM generating cell internal deformation, allowing it to evaluate the mechanical conditions of its environment. Thus, guided by the stiffness of the ECM, the cell generates new adhesions in the migration direction at the cell front and releases adhesions at its rear [47,48]. The forces generated by the *AM* motor depend on the internal deformation of the cell, which, in turn, depends on the ECM stiffness. In this model, the cell internal stresses, $\sigma_{cell}$, are defined through the cell internal deformations, $\varepsilon_{cell}$, considering the contributions of the active *AM* apparatus, as well as the cell passive elements (Figure 2a). Thus, the cell internal stresses can be calculated in each membrane node as [31,39,49].

(1) $$\sigma_{i}=\left\{\begin{array}{cc} K_{pas}\;\varepsilon_{i} & \varepsilon_{i}<\varepsilon_{min}\;\mathrm{or}\;\varepsilon_{i}>\varepsilon_{max}\;,\\ \frac{K_{act}\sigma_{max}\left(\varepsilon_{min}-\varepsilon_{i}\right)}{K_{act}\varepsilon_{min}-\sigma_{max}}+K_{pas}\;\varepsilon_{i} & \varepsilon_{min}\leq \varepsilon_{i}\leq \tilde{\varepsilon }\;,\\ \frac{K_{act}\sigma_{max}\left(\varepsilon_{max}-\varepsilon_{i}\right)}{K_{act}\varepsilon_{max}-\sigma_{max}}+K_{pas}\;\varepsilon_{i} & \tilde{\varepsilon }\leq \varepsilon_{i}\leq \varepsilon_{max}\;, \end{array}\right.$$

where $\sigma_{i}$ and $\varepsilon_{i}$ are the internal stresses and internal deformations at the *i*th node, respectively. $\sigma_{max}$ is the maximum stress that the *AM* is able to exert. $K_{act}$ and $K_{pas}$ correspond to the active and passive stiffness of the internal cell elements, respectively. Cell forces are defined in the range of $\varepsilon_{max}$ and $\varepsilon_{min}$, which is the strain range where *AM* is actively generating forces. $\tilde{\varepsilon }$ is the cell strain corresponding to the maximum forces, which is defined as $\tilde{\varepsilon }=\sigma_{max}/K_{act}$.

Cell internal deformations, $\varepsilon_{i}$, are calculated as the variation of the distance between any membrane node, $M_{i}$, and the cell centroid, *O*, as [42,49] (Figure 1c):

(2) $$\varepsilon_{i}=\frac{\Delta OM_{i}}{OM_{i}},\;\;i=1:n\;.$$

Cell traction forces depend on the internal stresses of the cell and on the number of cell adhesions, which, in turn, depend on the number of available receptors, $n_{r}$, and the cell ligand density, ψ. Thus, the nodal cell traction force, $F_{trac}^{i}$, is calculated for each membrane node as [38,50]:

(3) $$F_{trac}^{i}=\sigma_{i}\;S\;k\;n_{r}\;\psi \;e_{i}\;,$$

where *S* and *k* are the cell membrane surface and the binding constant, respectively. $e_{i}$ is the direction unit vector of the membrane node towards the cell centroid. Thus, the resultant traction force of the cell, $F_{trac}$, can be calculated by [41]

(4) $$F_{trac}=\sum_{i=1}^{n}F_{trac}^{i}\;.$$

We consider the protrusion forces, $F_{prot}$, due to the generation and retraction of protrusions in the cell membrane. These protrusions are considered as a cell random process, which induces the cell to move in random patterns. The magnitude of the protrusion forces is considered in the same order of magnitude as the traction force. Thus, the protrusion force is calculated by [39,51]

(5) $$F_{prot}=\kappa \left\|F_{trac}\right\|e_{rnd}\;,$$

where κ is a random parameter between 0≤κ≤1. $e_{rnd}$ is a random unit vector, which defines the direction of the protrusion force.

Additionally, cells are guided by the electrical stimulus (ES) through electrotaxis. Although the specific processes that guide cells through electrotaxis are still unclear, different studies have shown a relationship between the influx of $Ca^{2+}$, cell hyperpolarization, and depolarization [52]. Furthermore, the migration velocity seems to be proportional to the intensity of the ES until a saturation point is reached, for which the velocity is maintained even with the increase in the ES [53]. B. Frederich et al. observed that different cardiac cells show a proportional response to the electric field intensity [54]. Thus, we consider the electric forces, $F_{EF}$, due to the ES, proportional to the electric field, *E*, as [50,51]

(6) $$F_{EF}=\left\{\begin{array}{cc} -E\;\Omega \;S\;e_{EF} & E\leq E_{sat}\;,\\ -E_{sat}\;\Omega \;S\;e_{EF} & E>E_{sat}\;, \end{array}\right.$$

where *E* is the magnitude of the electric field. $E_{sat}$ corresponds to the maximum electric field, which shows a saturation of the electrical stimuli [53,54,55]. Ω is the cell surface charge density. $e_{EF}$ is the direction of the electric field.

Likewise, we consider the repelling electric forces produced by the presence of other charged cells in the ECM. Thus, the electric force, $F_{EF}^{ij}$, experienced by *i*th cell due to the electric repulsion generated by *j*th cell, which is proportional to the electric charge of the cells, $\Omega_{i}$ and $\Omega_{j}$, can be calculated by [50,51]

(7) $$F_{EF}^{ij}=\frac{k_{e}}{\epsilon_{r}}\frac{\Omega_{i}S_{i}\Omega_{j}S_{j}}{r_{ij}^{2}}e_{ij},$$

where $k_{e}$ is the coulomb constant, and $\epsilon_{r}$ is the relative permittivity of the ECM. The resultant electric force, $F_{elec}$, experienced by a cell is obtained as the sum of the contribution of the other *j*th cells and the electric forces generated by the ES as [50,51]

(8) $$F_{elec}=F_{EF}+\sum_{j=1}^{n-1}F_{EF}^{ij}\;.$$

Moreover, due to the movement of the cell on a viscous ECM, we consider the effect of the drag force. As the drag force, $F_{drag}$, is proportional to the cell velocity, v, and the ECM viscosity, η, it can be defined by [49,56]

(9) $$F_{drag}=f_{sh}\eta v\;,$$

where $f_{sh}$ is the shape factor of the cell, which is calculated as $f_{sh}=6\pi r$ for a quasi-spherical single cell [37,38,39]. Therefore, the resultant cell forces can be defined as

(10) $$F_{trac}+F_{elec}+F_{prot}=F_{drag}\;.$$

### 2.3. Cell Interaction

Cell–cell interactions play a key role in different cellular processes such as cell proliferation [57,58] and migration [59,60]. Through cell contacts, cells establish different processes of molecular communication, which are essential for collective cell migration [4] and different tissue-level processes [61]. In fact, cardiac tissue functionality depends on cell–cell interaction quality, which includes cell orientation and cell architecture [62]. To carry out an in vivo tissue-like structure, it is essential to understand and control cell–cell interactions. In this section, the implementation of cell–cell interactions and their collective response in the model is described.

Cell contact is defined, for any pair of cells (Figure 2b), considering cell-cell distance as

(11) $$X_{ij}=X_{i}-X_{j}\;,$$

where $X_{ij}$ is the contact vector that defines the distance between the *i*th and *j*th cells. It can be defined by the cells position vectors $X_{i}$ and $X_{j}$. Cell contact vector must fulfill $\|X_{ij}\|\geq 2r$ in order to avoid cell–cell superposition. In the particular case of $\|X_{ij}\|=2r$, cells are considered to be in contact (Figure 2b). In this case, stable cell adhesion is considered if the cell contact is properly oriented with respect to cell global polarization. Hence, the contact direction can be defined as (Figure 2c)

(12) $$e_{ij}=\frac{X_{ij}}{\|X_{ij}\|}\;.$$

In this case, cell polarization, $e_{pol}^{i}$, depends on the direction of the different stimuli which are acting on the cell. As the mechanical and electrical stimuli are considered in the present model, cell polarization can be defined by [39]

(13) $$e_{pol}^{i}=\frac{e_{mech}^{i}+e_{elec}^{i}}{\|e_{mech}^{i}+e_{elec}^{i}\|}\;,$$

where $e_{mech}^{i}$ and $e_{elec}^{i}$ are the direction of the mechanical and electrical stimuli, respectively. Thus, mechanical stimuli direction, $e_{mech}^{i}$, can be calculated as [49,50]

(14) $$e_{mech}^{i}=\frac{F_{trac}}{\|F_{trac}\|}\;,$$

and the electrical stimuli direction can be calculated as [40,49]

(15) $$e_{elec}^{i}=\frac{F_{i}^{EF}}{\|F_{i}^{EF}\|}\;.$$

Moreover, the direction of the global polarization, $G_{pol}$, is considered, which indicates the orientation of the majority of the cells (Figure 2c). N. Tahara et al. exposed that during cardiac cell migration, the cells tend to establish cell–cell attachments to form coherent epithelia in cardiac populations [63]. In fact, cardiac cells establish structured cell architectures to form tissues with anisotropic properties [64,65,66,67,68]. Thus, we define the direction of the global polarization, $G_{pol}$, which depends on the polarization of each cell, $e_{pol}^{i}$, as [39]

(16) $$G_{pol}=\frac{R_{pol}}{\|R_{pol}\|}\;,$$

where:

(17) $$R_{pol}=\sum_{i=1}^{n}\frac{e_{pol}^{i}}{\|e_{pol}^{i}\|}\;.$$

**Figure 3:**
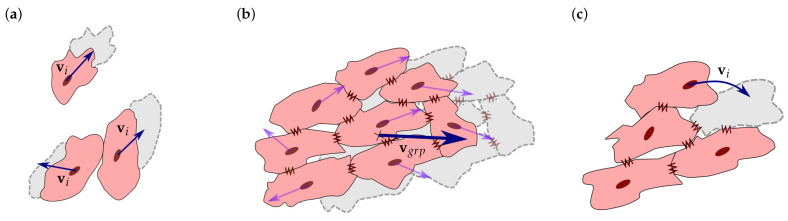
Cell migration and interaction. (**a**) Individual cell migration. $v_{i}$ is considered for individual cells and for cells that are not attached to any other cell. (**b**) Collective cell migration is considered for groups of cells that are attached by cell junctions. Group velocity, $v_{grp}$, is defined on the basis of the migratory tendency of the individual cells of the group. (**c**) Cell relocation is considered when group velocity is insufficient to consider group movement. Any internal cell can migrate to a more favorable position with its individual velocity, $v_{i}$, without detaching the group.

The $G_{pol}$ direction defines the global direction of the tissue fibers, which is related to the cell–cell stable adhesions. Thus, we establish a stable cell junction (CJ) between two cells in contact if the direction of the cell–cell contact, $e_{ij}$, is coherent with the direction of the global polarization, $G_{pol}$. For this purpose, we define the contact projection, $l_{ij}$, for each cell contact, as [39]

(18) $$l_{ij}=\frac{Proj(e_{ij},G_{pol})}{\|G_{pol}\|}\;,$$

where $l_{ij}$ has a value that satisfies $0<l_{ij}\leq 1$. Thus, if the contact direction is perpendicular to the $G_{pol}$, i.e., $l_{ij}=0$, there is no CJ. In contrast, if the contact direction is close or equal to the $G_{pol}$, i.e., $l_{ij}\geq l_{adh}$, the cells are considered to be attached by CJs (Figure 2c) [14,39,69,70]. As observed by C. Sassoli et al., CMs form clusters that migrate collectively [71]. Thus, once the cells are integrated into a group, a collective cell migration is considered. Unlike the individual cell migration (Figure 3a), cells attached by CJs tend to form stable groups and migrate collectively (Figure 3b) [71,72,73].

In this case, each cell in the group contributes to the movement based on its individual traction, $F_{trac}^{i}$, electric, $F_{elec}^{i}$, and protrusion, $F_{prot}^{i}$, forces. Thus, each cell pulls the group towards the direction where it wants to migrate. The drag force of the group is defined by the summation of the forces of all the group cells, which can be calculated as

(19) $$F_{drag}^{grp}=\sum_{i=1}^{n}F_{trac}^{i}+F_{elec}^{i}+F_{prot}^{i}\;.$$

Group velocity, $v_{grp}$, can be calculated through Equation (9). Due to the irregular shape of the group, the shape factor, $f_{sh}$, must be calculated considering the group’s geometry. Thus, it can be defined as [38]

(20) $$f_{sh}={\left[\frac{l_{max}l_{med}}{l_{min}^{2}}\right]}^{0.09}6\pi r_{grp}\;,$$

where $r_{grp}$ is the equivalent radius of the group. $l_{max}$, $l_{med}$, and $l_{min}$ are the maximum, medium, and minimum dimensions of the group, defined in an orthogonal local system, respectively.

Moreover, cells can migrate internally within the group to a more favorable position. In this case, to avoid duplicity of movements, if the group’s velocity is not enough to move the group, while an internal cell is able to move to a new position without leaving the group, this cell is relocated into that new position (Figure 3c) [39,71,74].

### 2.4. Cell Fate

Cell processes such as cell differentiation, maturation, proliferation, and apoptosis depend, among others, on the mechanical properties of the ECM. For instance, some studies have suggested that maturation rates are dependent on the ECM stiffness, in such a way that faster maturation has been observed in stiffer ECM [75,76,77]. In cardiac tissues, both electric and mechanical stimuli play a key role during tissue development. Besides, both stimuli are essential and needed for correct cell maturation [16]. In the present model, cell maturation is considered to be based on the mechanical stimulus perceived by the cell, which, in turn, depends on the cell’s internal deformation. Thus, we define the parameter $\gamma_{c}\left(t\right)$, which determines the intensity of the mechanical stimulus perceived by the cell at each time step, *t*, as [39]

(21) $$\gamma_{c}\left(t\right)=\frac{1}{n}\sum_{i=1}^{n}e_{i}:\varepsilon_{i}:e_{i}^{T}\;,$$

where $\varepsilon_{i}$ is the cell deformation evaluated at each *i*th membrane node, and $e_{i}$ is the direction vector from the *i*th node towards the cell centroid.

To describe cell maturation, we define $t_{mat}\left(\gamma_{c},t\right)$ as the time necessary by a cell to maturate and trigger cell processes such as differentiation and proliferation. Thus, $t_{mat}\left(\gamma_{c},t\right)$ can be determined by [51,56]

(22) $$t_{mat}\left(\gamma_{c},t\right)=t_{min}+t_{p}\gamma_{c}\left(t\right)\;,$$

where $t_{min}$ is the minimum time needed for cell maturation, and $t_{p}$ is a proportional time, which depends on the mechanical stimuli to which the cell is subjected. As the cells interact with the ECM, it is necessary to define the state of maturation of the cell, which is related to the cell-cycle evolution [78]. For this purpose, a Maturation Index (MI) is defined as cell-cycle completed time, which is calculated as [51,56]

(23) $$\mathrm{MI}=\left\{\begin{array}{cc} \frac{t}{t_{mat}} & t\leq t_{mat}\;,\\ 1 & t>t_{mat}\;. \end{array}\right.$$

As the MI represents the cell-cycle progress, it is considered that cells are able to trigger cell proliferation and/or differentiation, depending on the considered cell, when MI=1. Cell differentiation is considered for a stem cell, ST, when the cell is completely mature. In this case, the adopted cell phenotype depends on the mechanical stimuli, $\gamma_{c}$, perceived by the cell from the ECM. Furthermore, the mechanical stimulus perceived from an ECM with stiffness similar to that found in cardiac tissues has been shown to be capable of triggering CM differentiation [10,16]. Moreover, cell apoptosis is considered when the mechanical stimuli exceed a certain limit, $\gamma_{apop}$, which produces permanent damage to the cell [14,79]. Thus, cell phenotype, i∈{ST,CM}, is defined depending on the MI and the mechanical stimulus of the cell as

(24) $$\mathrm{Cell}\;\mathrm{state}=\left\{\begin{array}{cc} \mathrm{CM}\; & \gamma_{min}<\gamma_{c}\leq \gamma_{max}\;\&\;\mathrm{MI}=1\;,\\ \mathrm{apoptosis}\; & \gamma_{apop}<\gamma_{c}\;,\\ \mathrm{no}\;\mathrm{differentiation} & \mathrm{otherwise}\;, \end{array}\right.$$

where $\gamma_{min}$ and $\gamma_{max}$ correspond to the mechanical stimuli for the minimum and maximum ECM stiffnesses, respectively, which have been shown to trigger CM differentiation spontaneously.

Likewise, in the present model, cell proliferation has been considered for a mature cell. In general, cardiac cells have a low capacity for proliferation, which is related to the cell arrest due to functional assembly with other cells through CJs [78,80,81]. However, cardiac cell proliferation is related to phenotype maturity [78]. Thus, CM in the early stages of maturation (early CM) retains the capability of proliferation [78,82]. Once CM is attached to other cells, it develops an adult phenotype (late CM), where proliferation is inhibited due to the cell-cycle arrest [64,78,81,82,83]. In the present model, when there is no stable CJs, the model considers cardiac cells in the early stages of cardiac maturation (early CM), which retains proliferation capacities. Meanwhile, when there are stable CJs, the model considers cell proliferation inhibition due to cell–cell adhesion. Thus, early CM proliferation is regulated depending on the MI, which indicates its cell cycle state, and its adhesion with other cells, which is defined by the CJ. Then, cell proliferation is defined as

(25) $$\mathrm{Cell}\;\mathrm{proliferation}=\left\{\begin{array}{cc} 1\;\mathrm{mother}\to 2\;\mathrm{daughters} & {\mathrm{CJ}}_{i}<{\mathrm{CJ}}_{max}\;\&\;\mathrm{MI}=1\;,\\ \mathrm{no}\;\mathrm{proliferation} & \mathrm{otherwise}\;, \end{array}\right.$$

where ${\mathrm{CJ}}_{i}$ is the number of stable adhesions of the cell, and ${\mathrm{CJ}}_{max}$ is the maximum number of adhesions for which the cell-cycle arrest is promoted. This inhibition of cell proliferation is considered when at least 50% of the cell membrane has stable CJs. Due to the Finite Element discretization of the cell, the maximum possible stable adhesion for a cell corresponds to 8, consequently, ${\mathrm{CJ}}_{max}=4$ is defined. Cell proliferation generates two daughter cells from a mother cell. The daughters cells have the same phenotype and properties as the mother cell. The positions of these two new cells, $x_{daut}^{\left(1\right)}$ and $x_{daut}^{\left(2\right)}$, are defined as

(26) $$\begin{array}{cc} \; & x_{daut}^{\left(1\right)}=x_{moth}\;,\\ \; & x_{daut}^{\left(2\right)}=x_{moth}+2re_{rand}\;, \end{array}$$

where $x_{moth}$ is the position vector of the mother cell, and $e_{rand}$ is a random unit vector.

### 2.5. ECM Mechanical Behavior

The ECM, which gives structural and vital support to the cells, is usually made up of biocompatible hydrogels [84]. The stiffness of these hydrogels can be modulated depending on their composition as well as their mechanical conditions, making them highly versatile. For instance, the ECM may be reinforced by fiber or spherical piezoelectric material to enhance cell culture via mechanical and electrical stimuli [51,85]. In the present work, it has been considered that the ECM is composed of a hydrogel to which a rigid fiber has been added at the ECM center (Figure 1a). Moreover, this fiber has been considered as a material with piezoelectric (PZE) properties [86]. The ECM mechanical behavior has been considered as a linear elastic material, which can be simply described with linear stress–strain relationships as

(27) $$\sigma_{ij}=C_{ikjl}\epsilon_{kl}\;,$$

where σ and ϵ are the stress and strain tensors of the ECM, respectively. C is the fourth-order stiffness tensor of the ECM. The ECM has been considered as an isotropic material; thus, C can be defined through the Young’s modulus, *E*, and Poison’s coefficient, ν.

When the inserted fiber is considered, an active variation on the ECM stress–strain relation is produced. Besides, in the case of considering a PZE fiber, an electric field will be generated due to energy exchange between the generated strain energy and the electrical energy (Figure 1b). The constitutive equation for PZE materials can be defined through its stress tensor. Considering it as a linear elastic material, the stress tensor, $\sigma^{pze}$, can be defined as [51]

(28) $$\sigma_{ij}^{pze}=C_{ikjl}\left(\epsilon_{kl}^{pze}-g_{mkl}q_{m}\right)\;,$$

where $\epsilon^{pze}$ is the strain tensor of the PZE material. C, g, and q are the elastic stiffness tensor, the electric displacement vector, and the strain coefficient matrix, respectively.

The relationship between the stress tensor and the electric potential, E, generated by the PZE material can be expressed as [51]:

(29) $$q_{i}=D_{im}g_{mkl}\sigma_{ij}^{pze}+D_{ij}E_{j}\;,$$

where D is the dielectric properties of the material.

### 2.6. Electric Field Generated by the PZE Fiber

Electric field plays a key role in cardiac cell organization. It has been used during CMs maturation, showing an improvement in the contractile cell properties [16] as well as cell alignment [87]. PZE materials are capable of generating an electrical gradient when they become deformed. This property can be employed to generate an electrical cell stimulation in order to induce cells to migrate in the direction of the stimulus. Thus, we purpose a new ECM that includes a PZE fiber in the center of the longitudinal direction (Figure 1a). Once the ECM is deformed, an electric field is generated (Figure 1b).

**Figure 4:**
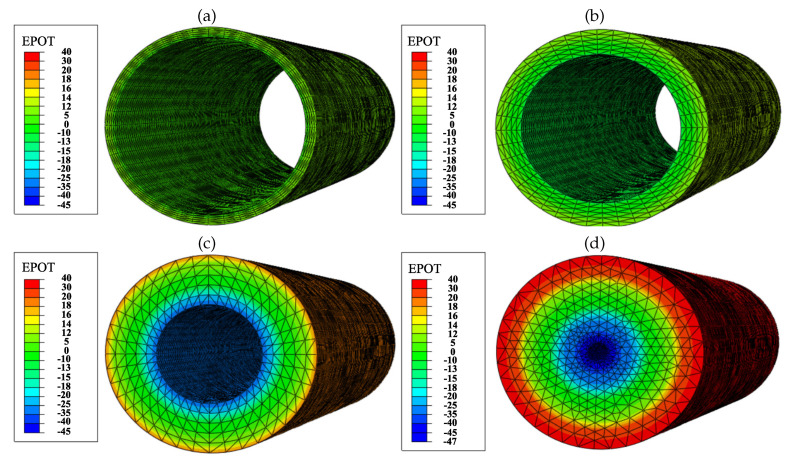
The electric potential (EPOT) [mV] generated by the PZE fiber with a longitudinal strain of 0.25. The electric potential has been evaluated for different wall thicknesses, corresponding to 2μm (**a**), 5μm (**b**), 10μm (**c**), and 18μm (**d**).

The PZE fiber has been evaluated when it is exposed to a deformation of 0.25, which is within the range of cardiac tissue deformation. The internal electric field generated by the PZE fiber depends on the applied strain and the fiber wall thickness. Thus, we evaluated the electric potential generated by the PZE fiber for wall thicknesses of 2μm (Figure 4a), 5μm (Figure 4b), 10μm (Figure 4c), and 18μm (Figure 4d). The outer surface of the PZE fiber is considered to acquire a positive charge, while the inner face becomes negatively charged. As the thickness of the material increases, the potential difference between the two faces increases. Thus, the generated electric field is homogeneous in the radial direction. In contrast, in the longitudinal and circumferential directions, the electric field gradient is negligible. Considering the electric charge of the ECM is kept neutral, the potential generated on the outer face of the PZE fiber generates an electric field in the range of 50–200 $\;V\;m^{-1}$. The minimum intensity of the electric field ($50\;V\;m^{-1}$) is obtained for the PZE fiber with the minimum thickness (2μm), and the maximum electric field ($200\;V\;m^{-1}$) is obtained for the PZE fiber with the highest thickness (18μm). This range of electric fields is within the range of those that can be found in the bibliography [17,54,87,88,89]. In this way, it is possible to combine the mechanical and electrical stimulation of the cells, by tuning the imposed deformation on the PZE fiber with its thickness.

### 2.7. Finite Element Model

The FEM has been employed to calculate the mechanical behavior of the different ECM configurations as well as cell behavior. The considered ECM has dimensions of 800×400×400μm. It has been discretized by trilinear hexahedral elements. A more rigid (PZE) hollow cylindrical fiber, with an external diameter of 40μm and a thickness in the range of 5–18μm, has been located in the center of the ECM. The considered fiber has been discretized with trilinear tetrahedral elements, if applicable, with PZE properties. An unconstrained ECM has been considered. Hence, minimum constrains are considered only to ensure calculation stability (Figure 1a). External longitudinal displacement is imposed to evaluate the effect of the change on the fibered ECM stiffness as well as the activation of the PZE material on the cells. This external displacement generates a deformation of the order of 25% in the fibered ECM, which produces an electric field gradient in the case of PZE fibered ECM (Figure 1b).

**Figure 5:**
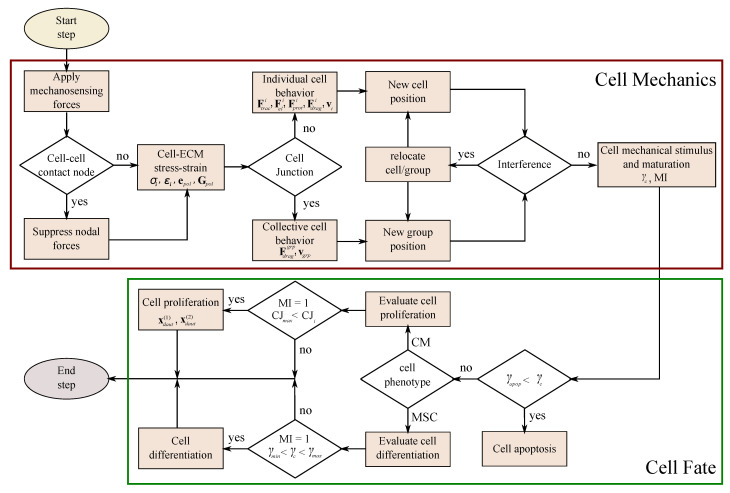
Algorithm of the model implemented for each time step.

The cell has been discretized as a quasi-spherical element defined by 24 nodes located in the cell membrane (see Figure 1c). This element has been implemented through a user-defined subroutine (UELMAT) in the commercial FE software Abaqus [43]. Cell forces are calculated in each membrane node, considering the stress–strain equilibrium on the cell–ECM interface. All the different cell processes are evaluated at each time step, as described in Figure 5. Each time step considers 1 h of cell–ECM interaction. The necessary model parameters are detailed in Table 1.

## 3. Numerical Examples and Results

**Figure 6:**
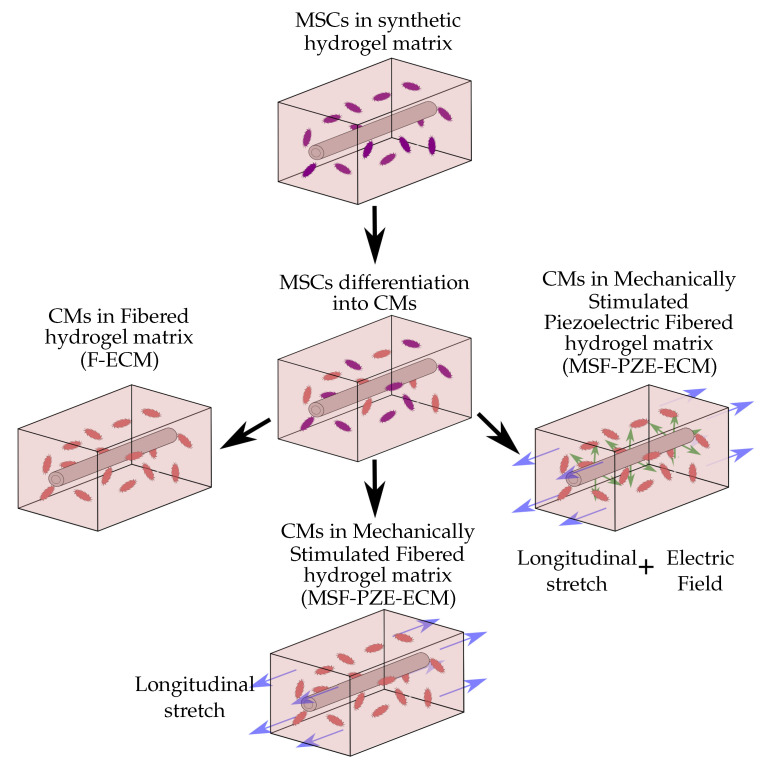
Schematic representation of the experiment setup. Cells are randomly seeded in a 20 kPa stiffness synthetic hydrogel matrix. Cell behavior has been studied under different stimuli, which include ECM stiffness, externally applied force, and electric field stimulus. For all cases, an initial phase of MSCs differentiation into MCs is considered.

To evaluate and compare the effect of the electrical and mechanical stimulation, different cases have been purposed and studied (see Figure 6). Since the differentiation phase is repetitive in all the cases, a separate study of cell differentiation has been carried out. Thus, in the first case, the differentiation of MSCs into CMs is presented. Subsequently, to focus on studying the effects of the different stimuli on cell behavior, different culture conditions are evaluated starting from differentiated cells. Therefore, the other three experiments of differentiated CMs have been elaborated. The first one is the simplest case, where differentiated cardiac cells are considered to be induced by the mechanical stimulus derived from the fibered ECM. Subsequently, the effects of the deformation of the composite ECM are studied (mechanically stimulated fibered ECM). Finally, the electro-mechanical effect due to the presence of the PZE material is considered (mechanically stimulated PZE fibered ECM). For this last case, the piezoelectric material has been independently evaluated (Section 2.6) to study the relationship between the deformation and the resultant electric field using the commercial FE software Abaqus [43].

### 3.1. Mesenchymal Stem Cells Differentiation into Cardiac Cells

#### 3.1.1. Description

Stem cells from different sources, such as MSCs, have been demonstrated to be a powerful tool to improve tissue regeneration [28,64,100,101]. MSCs retain the capacity to differentiate into different cell lineages, and they have a high proliferation rate, both essential for tissue development and regeneration [10,81]. Among others, ECM stiffness has been shown to be an effective stimulus to promote MSCs differentiation [8]. When the ECM stiffness is in the range of cardiac tissues (10–20 kPa) [16], it stimulates the MSCs to differentiate into cardiac cells [16,96,100]. Li et al. studied CMs differentiation from MSCs, by exposing them to ECM in the range of 16–65kPa stiffness. They observed an improvement in cardiac cell differentiation for ECM stiffness close to that of healthy tissue. Thus, they recommend cell differentiation in ECM stiffness below 50kPa [10]. In addition, Stoppel et al. concluded that stiffness higher than those which can be found in healthy tissues could inhibit CMs maturation [16]. In this sense, we studied MSCs differentiation and proliferation via a parametric model that depends on the cell mechanical stimulus.

#### 3.1.2. Experiment Setup

In this experiment, cell differentiation of MSCs into CMs has been studied. Sixty MSCs have been randomly distributed in a homogeneous hydrogel matrix with a stiffness of 20kPa with a central fiber of 25kPa stiffness. Cell interaction with the ECM was studied for 75 h. In this first case, neither mechanical nor electrical stimulation was applied, as cell differentiation is guided by the internal ECM stiffness.

#### 3.1.3. Results

**Figure 7:**
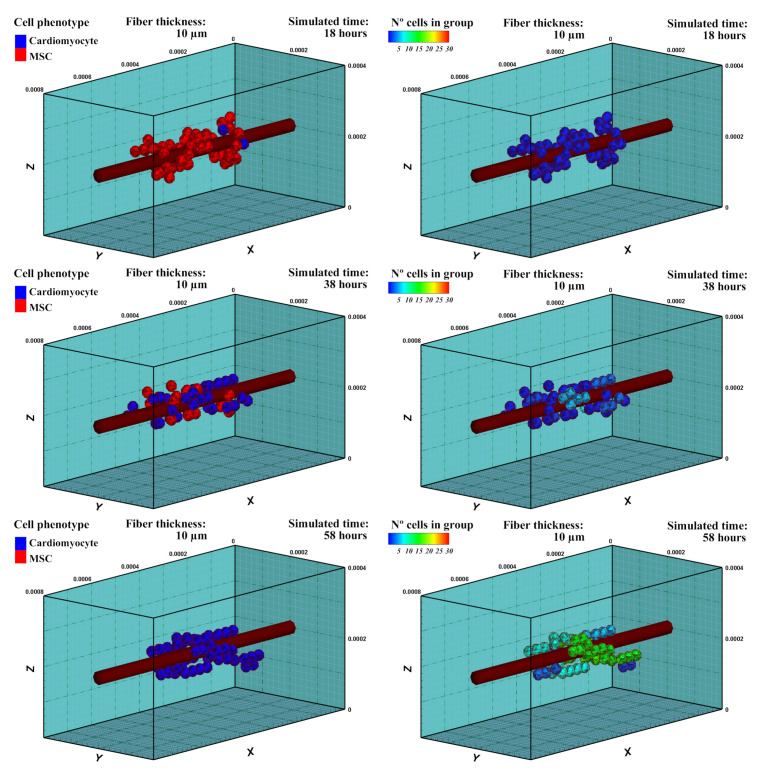
Cell phenotype (**left**) and group formation (**right**). Cell differentiation, induced by the mechanical stimulus, has been evaluated in an ECM of 20 kPa stiffness. After 18 h of stimulation, cells start differentiation into CMs. As cells adopt cardiac phenotype, groups with stable cell junctions start to form. After 38 h, groups of 3–5 cells can be observed. After 58 h, all the cells are differentiated into the cardiac phenotype. The number of cells of the groups are continually increasing with time (see also Supplementary Material: S1).

Cells evaluate the mechanical conditions of their surroundings, accumulating a mechanical stimulus during their maturation, which determines the adopted cell phenotype after a complete cell-cycle process [39]. After 18 h of cell culture, cells started to differentiate into cardiac cells (Figure 7), which is the considered cell phenotype in the subsequent experiment. It can be observed that MSCs differentiated throughout time with different maturation rates depending on the accumulated perceived mechanical stimulus of each cell. Hence, cell maturation was faster for cells that were close to the central fiber, which corresponds to the stiffest zone. During the MSCs phase, cells were able to form cell aggregations, but not a stable cell adhesion (CJ). As the cells differentiated into the cardiac cell phenotype, cell–cell interactions can promote cell stable junctions (CJ). After 38 h, some groups of CMs were observed (Figure 7). After 58 h of cell culture, all the cells differentiated into CMs. The new cells started to form stable groups with increasing numbers.

### 3.2. Fibered ECM

#### 3.2.1. Description

As it has been observed in previous works of our group [39,49], the ECM stiffness plays a key role in different processes such as differentiation, migration, and proliferation. Likewise, in experimental studies, cell behavior has been analyzed considering different mechanical stimulation generated by various mechanisms such as surface topography [65,102,103], medium geometry [104,105], and fibers orientation [70,106]. In this experiment, the effect of the presence of stiffer fiber in the ECM center (Figure 1a) on the formation of specific cellular architectures is studied.

#### 3.2.2. Experiment Setup

**Figure 8:**
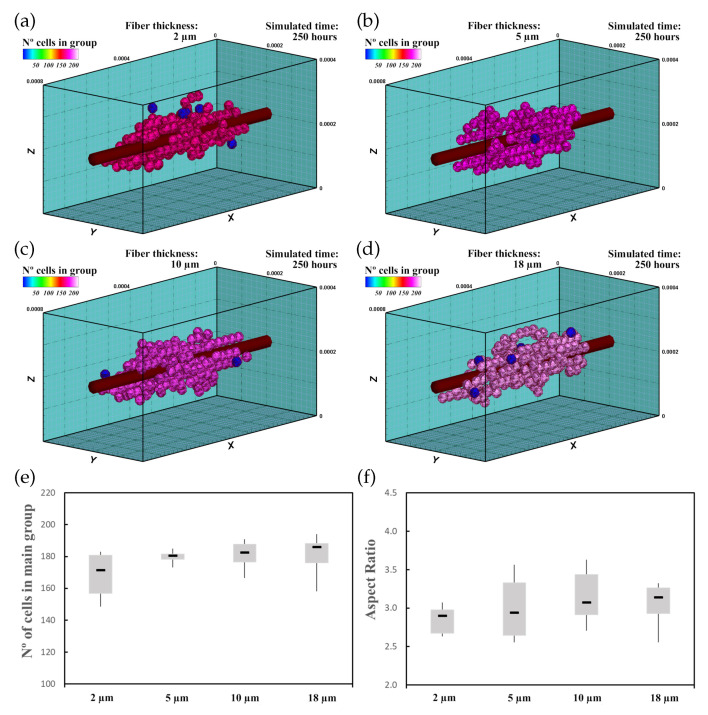
Group morphology and number of cells corresponding to an ECM with a central fiber thickness of 2μm (**a**), 5μm (**b**), 10μm (**c**), and 18μm (**d**). Cells migrate towards the central fiber where they keep moving and form the main group. As the fiber thickness increases, the mechanical stimulus is higher, which implies faster maturation rates and an increase in cell proliferation (see also Supplementary Material: S2). (**e**) Numerical results of the number of cells in the main group. (**f**) AR of the main group.

In this experiment, 60 randomly distributed differentiated cardiac cells were initially seeded in an ECM of 20 kPa stiffness that had a central fiber of 25 kPa stiffness, which is in the range of cardiac tissue stiffness [16]. The fiber had 2μm (Figure 8a), 5μm (Figure 8b), 10μm (Figure 8c), and 18μm (Figure 8d) thickness. This increase in fiber thickness implies an increase in the stiffness of the central zone. Cell behavior, including cell migration and group formation, was studied for 250 h. Considering the variability of the model, every experiment was repeated 10 times.

#### 3.2.3. Results

The cells, initially randomly distributed, migrated towards the center of the ECM, guided by the mechanical conditions as well as the stiffness of the central fiber. Upon reaching the central fiber, cells tended to remain in contact or move close to the central fiber. After 25–30 h, small groups of cells formed around the central fiber due to cell–cell interactions. They remained close to the central fiber due to its higher stiffness. After 250 h of culture, the increase in the size of the main group (at least 55% of the cells), due to the incorporation of new cells and the union of different groups, induced most of the cells to remain integrated with the main group. At the end of the simulation, one main group, formed by 160–180 cells, was established around the central fiber. As the cells joined the formed groups, cells reached the maturation state (late CMs), and consequently, cell proliferation considerably decreased.

To compare the group morphology, we define an Aspect Ratio (AR) parameter. On the basis of dimensions of the group, defined in an orthogonal coordinate system, the AR is defined as

(30) $$\mathrm{AR}={\left[\frac{l_{x}^{2}}{l_{y}l_{z}}\right]}^{0.5}\;,$$

where $l_{x}$, $l_{y}$, and $l_{z}$ are the longitudinal group length in *X* direction, and transversal group lengths in *Y* and *Z* directions, respectively.

The increase in fiber thickness implies an increase of the mechanical stimulus guiding cells to migrate toward the central zone. Thus, as the central fiber thickness increased, the cells tended to migrate faster toward the ECM center. Furthermore, an increase in maturation rate was observed as the fiber thickness increased, which consequently increased the proliferation rate of the cells (Figure 8e). In this context, as the fiber thickness increased, an increase in AR of the main group was recognized (Figure 8f), and groups with more elongated shape can be observed (Figure 8a–d).

### 3.3. Mechanically Stimulated Fibered ECM

#### 3.3.1. Description

Changes in the ECM mechanical conditions can alter cell maturation, reorganization, and alignment through the cell adhesion. For instance, the establishment of anisotropic conditions helps in the formation of muscular tissues [72]. In our previous work [39], we investigated the effect of the imposed strains on a fiber-free ECM with different stiffness on cell polarization and group geometry. The results show that the ECM deformation generates an increment in ECM stiffness in the deformation direction due to the residual forces. This could be an interesting way to control the alignment of cardiac cells to generate cardiac tissues with anisotropic properties.

#### 3.3.2. Experiment Setup

**Figure 9:**
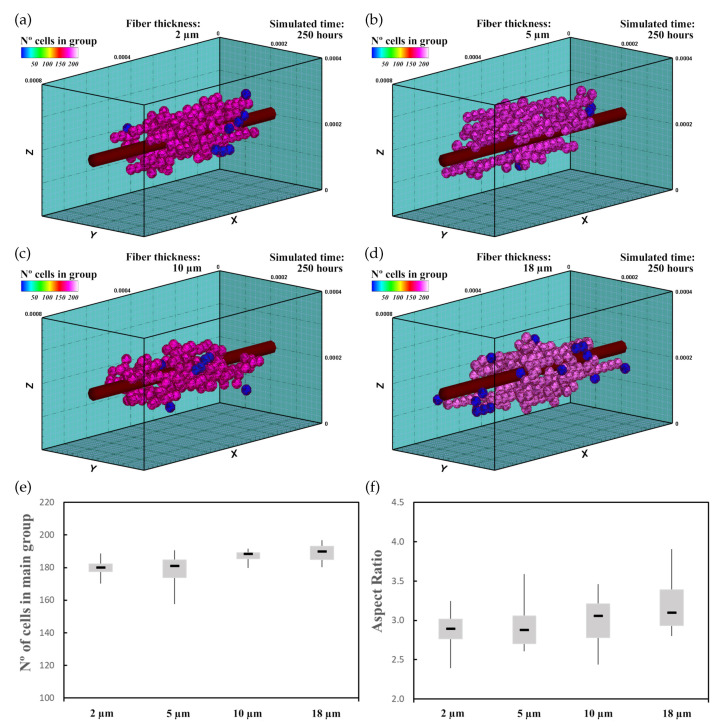
Group morphology and number of cells corresponding to an ECM with a central fiber thickness of 2μm (**a**), 5μm (**b**), 10μm (**c**), and 18μm (**d**). Cells are simulated in a deformed ECM where the passive deformation of the ECM is not considered on the cell deformation. Cells tend to migrate towards the central fiber where elongated groups are formed (see also Supplementary Material: S3). (**e**) Numerical results of the number of cells in the main group. (**f**) AR of the main group.

Thus, in the second experiment, 60 randomly distributed differentiated cardiac cells were initially seeded in an ECM of 20 kPa stiffness that had a central fiber of 25 kPa stiffness (Figure 9). In this case, a deformation of 0.25 was imposed in the longitudinal direction of the ECM (Figure 1a). Because of the applied deformation, residual forces in the longitudinal direction formed, which implies an increment in stiffness in this direction [39]. As in the previous case, fiber thickness varied for 2μm (Figure 9a), 5μm (Figure 9b), 10μm (Figure 9c), and 18μm (Figure 9d). Cell–cell and cell–ECM interactions were studied during 250 h of culture. The experiment was repeated 10 times for every case, with new initial random cell distributions.

#### 3.3.3. Results

The imposed deformation generated an anisotropic stiffening effect in the ECM. Thus, cells, guided by the mechanical stimulus due to the mechanical conditions as well as fiber stiffness increment tended to migrate to the central zone with higher velocity. Once there, cells started to form groups of a few cells that remained close to the central fiber. Due to the increase in stiffness, cells tended to stay closer to the central fiber, which reduced the tendency of cells to join the main group. This effect delayed the formation of the main group. Comparing with the previous case, interestingly, the number of cells at the end of the simulation increased (Figure 9e). In addition, the AR did not seem to increase significantly (Figure 9f). However, the relationship of the AR with the fiber thickness did not seem to be linear. After 250 h of culture, one main group formed around the central fiber of about 180–190 cells (Figure 9e), which was higher than in the previous case (Figure 8e). As the cells migrated faster to the ECM center, where the cell maturation was faster due to the higher mechanical stimulus, cell proliferation increased. As in the previous case, the main group geometry had an elongated shape aligned in the longitudinal direction.

### 3.4. Mechanically Stimulated PZE Fibered ECM

#### 3.4.1. Description

Electrical stimulation can guide cells in the direction of the electric field [54,87,89]. It has been seen that it improves cell maturation, contractile capacities, and alignment [16,17,54,66]. Although the effects of the mechanical and electrical stimulation can be beneficial, the study of the cellular response due to their coupling effect is not straightforward [40]. Thus, here, we studied the coupling of these effects on CMs behavior. Employing PZE fiber, the electrical stimulus, coupled with the mechanical deformation, was induced on the ECM, which generated a simultaneous and coordinated electro-mechanical stimulus.

#### 3.4.2. Experiment Setup

As in the previous cases, 60 differentiated cardiac cells were initially randomly distributed in 20 kPa stiffness ECM, with a central PZE fiber of 25 kPa stiffness. The PZE fiber thickness varied in the range of 2–18μm, and a longitudinal deformation of 0.25 was externally applied (Figure 1a). The initial passive deformation of the ECM was not considered in the calculation of the cell deformations given the ability of the cells to adapt themselves to the new situation. The deformation of the PZE fiber generated a homogeneous electric field in the radial direction in the range of 50–200 $\;V\;m^{-1}$ (Figure 4). This field stimulated the migration of CMs towards the central fiber coupling its effects with the mechanical stimulation. The cell–cell and cell–ECM interactions were observed during 250 h of culture. The experiments were repeated 10 times for every case.

#### 3.4.3. Results

**Figure 10:**
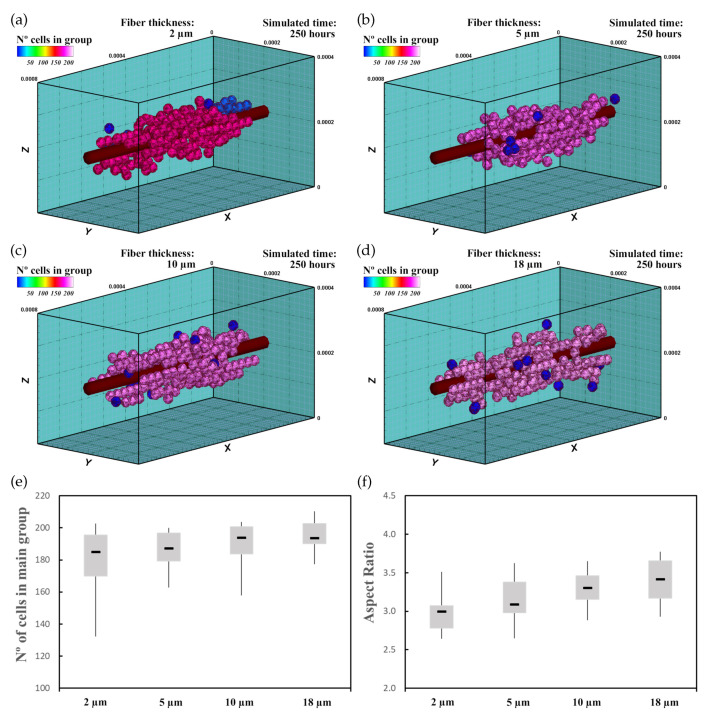
Group morphology and number of cells corresponding to an ECM with a central fiber thickness of 2μm (**a**), 5μm (**b**), 10μm (**c**), and 18μm (**d**). PZE fiber generates an electric field in the range of 50–200 $\;V\;m^{-1}$, which depends on the fiber thickness. The mechanical and electrical combined effect induces faster cell migration towards the central fiber where cell proliferation increases. After 250 h, cells form an elongated group around the central fiber (see also Supplementary Material: S4). (**e**) Numerical results of the number of cells in the main group. (**f**) AR of the main group.

As the PZE behavior was considered, cells were stimulated by an electric field of $50\;V\;m^{-1}$, $75\;V\;m^{-1}$, $100\;V\;m^{-1}$, and $200\;V\;m^{-1}$ (Figure 9a–d) corresponding to fiber diameters of 2μm, 5μm, 10μm, and 18μm, respectively. This stimulus, added to the mechanical stimulus, reduced the time that the cells needed to reach the central fiber. Once the cells reached the central fiber, the cells remained close to it and began to form small groups. The number of cells of these groups grew due to the integration of new cells. Then, these groups joined each other to form one main group. However, compared with the previous cases, this effect was slightly slower. This can be attributed to the overlapping effect of the electrical and the mechanical stimulus. After 250 h of culture, cells joined in one main group around the central fiber (Figure 10). The number of cells at the end of the simulation was higher than the previous cases, while the variability in the results seemed to be increased (Figure 10e). As the cells migrated faster to the center of the ECM, where the cell maturation was faster due to the mechanical stimulus, cell proliferation increased. As in the earlier cases, the AR of the formed groups increased as the thickness of the PZE fiber increased (Figure 10f). Compared with the previous cases, for the minimum fiber thickness, only a slight improvement was observed. However, as the fiber thickness increased, the AR increased considerably. In this case, the relationship of the AR with the PZE fiber thickness seemed to follow a linear tendency.

## 4. Discussion

Electrical and mechanical stimulation has been demonstrated to have relevant effects during the development of cardiac tissues. They can increase the functional maturity of the developed tissues. In addition, tissue contraction stresses depend, among others, on the capacity of the cells to establish an adequate cell internal structure, with well-organized sarcomeres, cell–cell communications, and aligned ultrastructure at tissue level [16]. In this way, the mechanical stimulus has been shown to have a close relationship with the increase in tissue maturation, with benefits in contractile machinery and hypertrophic pathways [16]. Additionally, an improvement in the contractile properties of CMs under electrical stimulation has been reported with an increase in cell–cell alignment, cell contractile machinery maturation, and calcium signaling [16,17,54,66]. B. Frederich et al. studied cardiac cells under electrical stimulus by exposing them to continuous electric fields. They observed an increase in the cells’ directionality during cell migration as the electric field increased [54]. S. Pietronave et al. have concluded that cardiac cells under electrical stimulus tend to increase cell alignment and adopt elongated cell shapes [87]. However, when electric fields are applied during long periods, the results show an increase in cell apoptosis. In fact, electric fields higher than 340 $V\;m^{-1}$ show also cell detachment and cell apoptosis [54,88].

The balance of the mechanical and electrical stimuli seems to be relevant to achieve the desired tissue properties. For instance, H. Heidi Au et al. cultured CMs under electrical and mechanical stimulation in chips with topographical cues (microgrooves with different depth). Their results show that the cell alignment was determined by the mechanical stimuli, while the electric field did not show a significant effect on the cell alignment [107]. Thus, in this direction, computational models can be helpful in studying and establishing preliminary configurations to balance the effect of the different stimuli.

In this paper, we present a new computational 3D model that includes the different relevant cell processes, such as cell migration, maturation, differentiation, proliferation, and cell–cell interactions. In it, different stimuli are taken into account. Hence, the coupling of different stimuli can be studied (Figure 6). In addition, the interaction of the cell with its complex ECM, where the mechanical and electrical cues have been coupled through the addition of a PZE fiber, has been taken into account. The model has been employed to study cardiac cell maturation. Thus, we purpose different experiment configurations, where cell behavior is studied. We started with MSCs differentiation into a CMs promoted by the mechanical stimulus of a fibered ECM. Then, we evaluated the CMs’ behavior and group formation under the effect of the mechanical stimulus into a fibered ECM. Afterwards, in the next experiment, an axial deformation of 0.25 has been added to the previous case. In the last case, the inner fiber the ECM of the former case has been considered to have PZE properties. Through these experiments, we have studied cell behavior, including processes such as differentiation, migration, and cell–cell and cell–ECM interactions as well as group formation and behavior.

**Figure 11:**
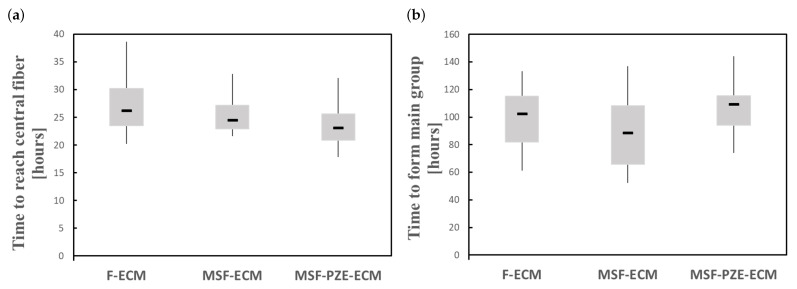
Numerical results under different stimulus configurations: Fibered ECM (F-ECM), Mechanically Stimulated Fibered ECM (MSF-ECM), and Mechanically Stimulated PZE Fibered ECM (MSF-PZE-ECM). (**a**) Time to reach the central fiber. (**b**) Time to join one main group (at least 55% of the cells).

In the case of fibered ECM (F-ECM), cells migrate toward the central fiber guided by the fiber stiffness, which is related to the increase in the mechanical stimulus. Meanwhile, in the case of Mechanically Stimulated Fibered ECM (MSF-ECM), the effects of the mechanical stimulus due to the residual forces generated by the imposed deformation are coupled to the effects of the fiber stiffness. The imposed deformation generates more stiffening to the central fiber as well as the ECM, which increases the velocity at which the cells migrate towards the central zone (Figure 11a). In addition, as the cells are in the stiffer zone for a longer period of time, cell maturation is faster, which increases cell proliferation. In the last experiment, an electrical stimulus is coupled with the previous stimuli via Mechanically Stimulated PZE Fibered ECM (MSF-PZE-ECM). In this case, the electrical stimulus has the same direction as the mechanical stimulus of the previous two cases. This coupled effect increases the velocity to migrate towards the central fiber (Figure 11a). As the electrical stimulus increases, cell migration toward the central fiber is faster. This is consistent with the bibliography, where cell response is proportional to the electric field intensity [54,87]. Moreover, as the stimuli towards the central fiber are increased, cells tend to keep near to the central fiber, reducing cell motility and preventing longitudinal migration, which delays the formation of the main group (Figure 11b). While the cells are kept for a longer time in the central zone, which corresponds to the most rigid zone of the ECM, the maturation is faster, and the proliferation increases. Increasing the thickness of the central fiber intensifies both the effects of mechanical and electrical stimuli. Thus, it accelerates the cell migration to the central zone. Consequently, the proliferation is faster (see Figure 8e, Figure 9e and Figure 10e).

As it has been observed, group morphology seems to have a strong relationship with the thickness of the central fiber. Thus, as the fiber thickness increases, the AR is higher for all the proposed cases. Likewise, a slight increase in the AR has been observed when PZE properties have been considered. When the electrical stimulus is coupled to the fiber stiffness stimulus, cells migrate faster to the central fiber, where they are kept close, with reduced motility. As cell motility is reduced, group formation is delayed. Consequently, the cell proliferation process continues for a longer time. Therefore, cell proliferation is increased compared with the previous cases. In this case, early groups are formed with a high degree of cell alignment in the longitudinal direction. In course of time, new cells and groups are incorporated to form the main chain. As the cells tend to keep in contact with the central fiber, due to the strong coupling of the mechanical and electrical stimuli, cell–cell interaction tendency in the longitudinal direction is increased. In this way, when the main group is formed, the degree of alignment is higher than in the previous cases.

On the other side, the present model has some simplifications and limitations that should be taken into consideration. For instance, cell morphology and behavior have been simplified, including, among others, the consideration of quasi-spherical cell morphology and the simplification of the molecular cell processes such as cell-cycle inhibition and molecular cell expressions. Moreover, the ECM has been considered as a homogeneous linear elastic hydrogel, neglecting the ECM cell remodeling. Despite these simplifications, the obtained results are qualitatively consistent with the literature, and interesting conclusions can be obtained. The simplicity of the model permits a better understanding of the complex cellular processes, and it allows the evaluation of a wide range of different culture conditions, with a low time and economic cost. In this sense, it can be helpful to establish the preliminary conditions in the experimental tests.

## 5. Conclusions

We have presented a new computational model to study CM behavior, which includes processes such as cell migration, maturation, differentiation, proliferation, and apoptosis in 3D-enhanced, PZE fibered matrices. The model has been employed to study cell–cell interactions during the formation of structured groups. The FEM has been employed to study the complex behavior of composite ECM as well as the cell response to the received stimuli. In this model, cells have been guided by a coordinated combination of mechanical and electrical stimulation through the consideration of a stiffer PZE fiber. To evaluate the response of the cell on different ECM configurations, different cases have been purposed (F-ECM, MSF-ECM, and MSF-PZE-ECM) (Figure 6).

Cells tend to migrate towards the stiffer zones (central fiber) guided by the mechanical and electrical stimuli proportional to the intensity of every stimulus [54,108]. The mechanical stimulus can be controlled through the application of longitudinal deformation and/or the variation of the fiber thickness. Whereas, through the PZE fiber, the electrical stimulus variation is associated to the mechanical stimulus. The results show that the coupling of mechanical and electrical stimuli can be a powerful tool to control CM essential processes. For instance, as the cells are in the stiffest zone close to the central fiber for a longer time, cell maturation is faster, which consequently improves cell proliferation. Hence, better results are obtained for the maximum PZE fiber thickness, which corresponds to the maximum mechanical and electrical stimuli ($200\;V\;m^{-1}$).

In conclusion, the longitudinal orientation of cell groups is increased as the stiffening effect of the central fiber increases. In this sense, stiffer fibers can be used as an anchor point that guides cells during in vitro tissue development. Moreover, cells can be guided by the electric field generated by the piezoelectric material. Thus, coupling the electric and mechanic stimulus with piezoelectric materials can be useful to control cellular architectures during in vitro tissue development.

Although some aspects, such as cell morphology, have been simplified, the obtained results are qualitatively consistent with the literature [17,18,54,74,87,89,108,109]. The authors believe that this model can be an effective tool to support the experimental models, being able to establish preliminary results to calibrate in vitro and in vivo experiments. Using such a computational model, which can predict cardiac cell behavior with reduced economic and temporary cost, can reduce dramatically the number of experimental assays. In such cases, computational methods can be considered as a helpful tool to adequately calibrate the mechanical and electrical stimuli, reducing the in vitro and in vivo experimentation.

## Figures and Tables

**Table 1:** Considered mechanical parameters in the model.

Parameter	Description	Value	Ref.
$K_{pas}$	Stiffness of the cell passive elements	2.8 kPa	[90,91]
$K_{act}$	Stiffness of the actin-myosin machinery	7.0 kPa	[90,92]
$\varepsilon_{max}$	Maximum strain of the cell	0.09	[38,93]
$\varepsilon_{min}$	Minimum strain of the cell	−0.09	[38,93]
$\sigma_{max}$	Maximum contractile stress exerted by the actin-myosin machinery	0.25 kPa	[94,95]
ν	ECM Poisson ratio	0.4	[96,97]
η	ECM viscosity	1000 Pa·s	[30,77]
*k*	Binding constant of the cell	$10^{8}$ mol${}^{-1}$	[30,51]
$n_{r}$	Number of available receptors of the cell	$1.5\times 10^{5}$	[30,51]
$E_{sat}$	Saturation value of electric field	$1200\;{\mathrm{Vm}}^{-1}$	[54]
$\Omega_{sat}$	Saturation value of cell charge density	$5^{-2}\;{\mathrm{Cm}}^{-2}$	[98,99]
ψ	Cell ligand concentration	$10^{-5}$ mol	[30,51]
$l_{adh}$	Minimum projection bound to consider cell adhesion	0.50	[70]
$t_{min}$	Minimum time needed for maturation	6 days	[64,81]
$t_{p}$	Time proportionality	200 days	[79,90]
$\gamma_{min}$	Minimum mechanical stimuli for cardiac cell differentiation	−0.04	[10,16]
$\gamma_{max}$	Maximum mechanical stimuli for cardiac cell differentiation	−0.01	[10,16]
$\gamma_{apop}$	Maximum mechanical stimuli that trigger apoptosis	0.6	[14,79]

## Supplementary Materials

The following are available at https://www.mdpi.com/2079-7737/10/2/135/s1, Video S1: SupplVid01.mp4, Video S2: SupplVid02.mp4, Video S3: SupplVid03.mp4, Video S4: SupplVid04.mp4.
